# Hospital World

**Published:** 1911-03-18

**Authors:** 


### THE HOSPITAL WORLD.

### Personalia.

MR. RICHARD FOSTER, of Homewood, Chislehurst, Kent, for some years one of the treasurers of the London Hospital, who died on December 23, aged eighty-eight years, left estate of the gross value of £188,919 19s. 3d., of which the net personalty has been sworn at £183,931 15s. 11d.

THE appointment of Mr. Mervyn King as vice-president of the Bristol General Hospital in place of the late Lord Winterstoke is a singularly appropriate one. Members of the King family have served the Redcliff Ward, in which the Bristol General Hospital is situated, for very many years. In Hunt's Bristol Directory of 1850, for example, the names of both the father and the uncle of the new vice-president of the Bristol General Hospital (Richard J. Poole King and William Poole King) are to be found as Councillors of Redcliff Ward, and the firm of "R. and W. King, African merchants, behind 2 Redcliff Parade," is also mentioned. From Redcliff Parade to the site of the Bristol General Hospital is but a stone's throw, and Mr. Mervyn King has taken advantage of this proximity to identify himself closely with the institution whose interests he has served for a number of years with far more than an ordinary degree of enthusiasm. The venerable president, Mr. Joseph Fry, who is now nearly ninety, but who still continues to afford personal service to his generation, had good reason for referring to Mr. Mervyn King's frequent visits to the hospital and his personal interest in its general welfare and in the staff. It would indeed have been hard to find another Bristol citizen so eminently calculated to occupy Mr. King's new position. Mr. Mervyn King shares with Mr. Hare the honour of being the oldest member of the House Committee. Everyone knows him as a friend, and the treasurer in the additional capacity of a generous donor.

### Elections and Appointments.

LORD MILNER, Sir Anderson Critchett, Sir R. Filmer, Sir Marcus Samuel, Sir Philip H. Waterlow, Colonel E. T. Luck, Colonel C. E. Warde, M.P.; Sir Reginald McLeod, Mr. C. H. Wheeler, M.P.; Mr. Alfred Austin, Mr. R. J. Balston, Mr. Charles Stewart Hardy, Mr. E. W. Hussey, and Mr. H. Monckton are the newly-appointed vice-presidents of the Kent County Ophthalmic Hospital.

EARL FITZWILLIAM has been re-elected President, and Captain Behrens chairman, of the Malton Cottage Hospital.

MR. W. W. HEWITT has been re-elected President of the Essex County Hospital.

CAPTAIN G. C. TRYON, M.P., has been elected President of the Brighton, Hove, and Preston Dental Hospital.

MR. JAMES BUCHANAN has been elected President of the Royal Sussex County Hospital, and the Bishop of Lewes, Mr. Frank Wisden, and Mrs. Walter Willett vice-presidents, Major Buttanshaw, Mr. R. G. Modera, Captain Butler, Colonel Cavenagh, Mr. R. W. Partridge, and Mr. Frank Wisden are to serve on the Board of Management for three years as elected members.

THE EARL OF PLYMOUTH has been re-elected President of the Redditch Smallwood Hospital, Birmingham.

MR. C. HYDE has been elected President; the Lord Mayor (Alderman Bowater), Alderman Lloyd, and Mr. W. S. Harding vice-presidents; Mr. J. W. Tilley, treasurer, and Mr. E. K. Kannreuther as temporary secretary of the Birmingham and Midland Free Hospital for Sick Children. Mr. C. Hyde, Mr. J. R. H. Smyth, and Mr. H. F. Harvey have been elected honorary life governors of the hospital.

